# Enhancing grid stability using dynamic reserve power point tracking techniques

**DOI:** 10.1371/journal.pone.0349249

**Published:** 2026-06-01

**Authors:** Sajjan Kumar, G. R. Venkatakrishnan, R. Rengaraj, Amit Kumar, Pankaj Kumar

**Affiliations:** 1 Sri Sivasubramaniya Nadar College of Engineering, Chennai, India; 2 Sershah Engineering College, Sasaram, Bihar, India; 3 Manipal Institute of Technology, Manipal Academy of Higher Education, Manipal, India; Aalto University, FINLAND

## Abstract

Grid stability is of prime importance for grid-tied solar power systems as they are prone to power quality issues caused by the varying intensity of sun radiation and grid disturbances. To maintain grid stability, various PV power tracking algorithms have been developed. However, classical power tracking models often fail to maintain grid stability and sustain required power reserves under real-time variations in grid conditions and solar generation. To address this limitation, a Dynamic Reserve Power Point Tracking (DRPPT) control algorithm is proposed to ensure grid stability by dynamically adjusting reserve power. By continuously monitoring the PV array and grid conditions, the proposed controller determines the dynamic solar reserve power and accordingly selects the suitable operating mode. The operating point of PV array is then regulated by Flexible Power Point Tracking (FPPT) technique, which performs fine-tuning of the reference voltage followed by grid injection. By combining FPPT and Maximum Power Point Tracking (MPPT) functionalities, the proposed DRPPT controller maintains optimal reserve levels, ensuring the grid can rapidly respond to sudden changes in power supply or demand while meeting customer requirements. The proposed model is tested on hardware and simulated on software, and both results show the ability of DRPPT algorithm to handle real-time grid frequency changes and adapt the RPPT operation accordingly to meet grid stability standards. The proposed model has achieved superior THD mitigation, thereby improving grid stability, with 53.75%, 50%, and 7.5% lower THD compared to the conventional RPPT, Global FPPT (GFPPT), and Genetic Algorithm (GA)-based FPPT techniques, respectively.

## 1. Introduction

Solar energy is one of the alternative sources of renewable energy, and if harvested properly, it will be adequate to handle the rising demand for electricity [1]. The electricity produced from solar power is dependent on temperature, irradiation levels, and the voltage generated in the PV module. This makes the output power change with irradiance and temperature, affecting the efficiency of the solar power system [2]. Therefore, Maximum Power Point (MPP) has to be tracked from the solar panel profile to generate maximum output power, irrespective of the changing weather conditions [3]. An algorithm called MPP Tracking (MPPT) is widely used to track the operating point from where maximum power can be extracted from the PV cell. Most of the MPPT techniques change the operating point of the system by regulating the duty cycle of the DC-DC converter [4].

MPPT algorithms are essentially used to maximize available solar power; however, they do not determine the PV power needed for grid injection to ensure grid stability [5,6]. This creates serious issues to the reliability and stability of the grid-connected solar power systems [7,8]. To reduce the effect of high solar power injection into the grid, power system operators are continuously updating the standards for the effective connection of solar power systems to the grid [9]. This includes various ancillary services to be supported by the PV systems, such as reliability enhancement, frequency response, voltage support, and efficiency improvement [10]. To provide these ancillary services, traditional MPPT controllers are getting replaced by RPPT control algorithms, which operate below the MPP and keep some amount of reserve power [11,12].

RPPT performs both Global Maximum Power Point (GMPP) tracking and grid power injection regulation simultaneously [13,14]. Based on which, the power reserve level is determined, which aids in damping transient disturbances, voltage oscillations, and frequency deviations occurring in the grid-side [9,10]. In addition, RPPT performs well even in partial shading scenarios by the continuous monitoring of PV curve at high frequency, thereby preventing the power system from getting trapped in local maxima problem [15]. Conventional FPPT techniques run at fixed curtailed powers, but RPPT techniques regulates power reserves dynamically according to grid conditions, offering a more stable and responsive grid-support mechanism, which makes RPPT better than FPPT [15,16]. Since limited research has been carried out on RPPT algorithms, a DRPPT has been proposed as a state-of-the-art control technique for PV power plants to improve grid stability. The major contributions of the proposed RPPT are,

- The primary aim of the proposed DRPPT algorithm is to ensure grid stability by effectively switching between FPPT and MPPT functionalities, thereby achieving both grid support and solar utilization.
- The proposed DRPPT algorithm constantly monitors grid voltage and frequency, and dynamically controls the solar power reserve in real time by absorbing or releasing reserve power during voltage or frequency disturbances.
- The proposed algorithm monitors the PV power–voltage curve at high frequency, ensuring accurate identification of the GMPP even under complex partial-shading patterns, thereby preventing trapping at local maxima.
- The proposed algorithm is validated based on THD mitigation, grid stability, power output, and reserve power tracking accuracy compared to existing power tracking techniques.

This research paper is sectioned as: Section 2 contains literature survey on various power tracking algorithms; Section 3 explains the proposed methodology; hardware and simulation results along with comparative assessment are provided in Section 4 and Section 5 has the conclusion of this paper.

## 2. Literature survey

In 2022, Narang et al. [17] suggested an RPPT technique to ensure grid stability and enhance power management in grid-tied PV plants. The suggested technique optimally regulates power extraction and supports grid demands under changing load conditions. It is highly feasible for to be implemented in advanced smart grid-PV systems.

In 2023, Tafti et al. [18] developed a Global FPPT (GFPPT) method to reduce the effect of partial shading in PV systems, resulting in improved performance under changing irradiance levels. The research emphasizes the drawbacks of conventional MPPT methods under shaded situations and advances an enhanced tracking mechanism to overcome the same. The experimental results verify the capability of the developed method to improve energy harvesting under complex shading conditions.

In 2024, Hamid Ouatman and Nour-Eddine Boutammachte [19] implemented a Genetic Algorithm (GA)-based FPPT algorithm to effectively enhance the output power and tracking accuracy considering fluctuation in solar irradiance and partial shading. The output presented in the paper shows significance of optimization algorithms in FPPT.

Zhong., et al. in 2021 [20] suggested a Power Reserve Control (PRC) technique to constantly and rapidly track power reserve ratio, even under changing irradiance levels. The suggested PRC achieves 50 ms tracking speed and less than 1% tracking error. However, the frequency response is not improved by the PRC technique.

Gomez-Merchan., et al. in 2020 [21] designed a Binary Search-based FPPT (BNS-FPPT) control algorithm to provide the necessary support to the main grid. Even though the BNS-FPPT algorithm minimized power tracking error with minimal oscillations, its performance is not enhanced under partial shading scenarios.

Pawar., et al. in 2021 [22] developed a Grid-Forming Control (GFC) model to maintain power reserve without MPP tracking in real-time. The developed model achieves voltage and frequency support to grid through inverters. Even though the Grid-Following (GFL) inverters provide grid ancillary support during disturbances, it has reduced performance on weaker grids.

Kumaresan., et al. in 2021 [23] presented a secant method integrated with FPPT algorithm to continuously regulate the PV system operating point for reaching the optimal power reference. The presented model exhibits faster convergence rate, high accuracy, reduced error, and minimal steady-state oscillations; however, it is not validated in real-time.

Chen., et al. in 2026 [24] developed frequency containment and spinning reserve services for balancing renewable energy production. A progressive McCormick envelope relaxation technique is employed to convexify hydrogen-gas blending flows. Evaluation results depict that the developed model lessens the total system operating cost.

Feng., et al. in 2025 [25] implemented a Benders-Combined Constrained Markov Decision Process (BC-CMDP) technique to suppress power generation and load demand imbalances. The implemented technique combines safe reinforcement learning and logic-based Benders decomposition to effectively mitigate the hazards associated with frequency instability. The BC-CMDP’s nonconvex policy optimization is handled by a natural policy gradient primal-dual optimization. Evaluation performed on the IEEE 118-bus network ensures the global non-asymptotic convergence performance of the implemented technique.

Fei., et al. in 2025 [26] presented a weather routing-based mechanism for a Multi-Energy Ship Microgrid (MESM) to optimize the speed and voyage route under dynamically changing weather conditions. A case study is performed on a real cruise voyage, and the results exhibit enhanced computational efficiency due to the integration of a Tailored Progressive Hedging (TPH) algorithm.

According to the existing researches, various power tracking techniques have been developed to maintain power reserve levels for maintaining grid stability. However, the existing RPPT/FPPT techniques mainly focus on maintaining fixed reserve levels rather than dynamic maintenance. They respond slowly to real-time grid disturbances and changing irradiance levels, resulting in insufficient grid support and higher THD values. In addition, they primarily focus on maximum power extraction rather than reserve power management. Moreover, they have no seamless integration with SCADA systems for supervisory control. To overcome this, a DRPPT model is proposed enhance grid stability even under changing conditions.

## 3. Proposed methodology

**Fig 1:**
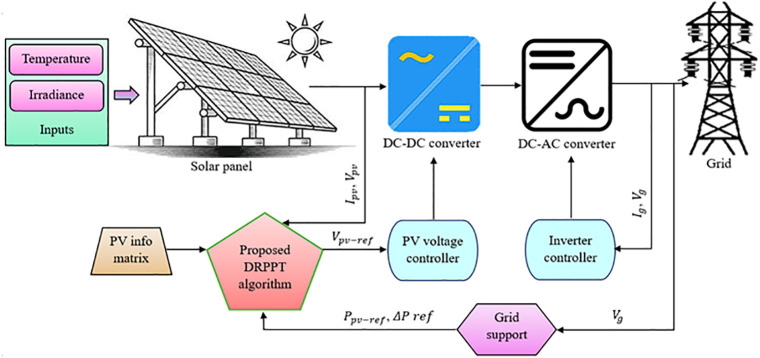
Block diagram of the proposed DRPPT controller.

In this section, the proposed DRPPT algorithm to ensure grid stability is discussed followed by the block diagram provided in Fig 1. The voltage (V_pv_) and current (I_pv_) generated by PV array is given as inputs to both the DC – DC converter and the proposed DRPPT controller. Then the proposed controller regulates the current and voltage, and sends the control action to the converter. The converter then steps up or downs the output voltage (V_o_) and current (I_o_) connected across the load. This controlled DC output is then given to the subsequent inverter stage, which plays a crucial part in grid interaction by converting the DC power into AC power and injecting it into the power grid. The proposed DRPPT controller, DC–DC converter, and inverter works in an integrated manner such that the PV system extracts optimal solar energy efficiently and supports various grid services, such as frequency response, voltage stabilization, and power reserve allocation.

### 3.1. Proposed DRPPT algorithm

RPPT is an advanced technique implemented in solar power plants that aids in extracting optimal solar power, while supporting grid by maintaining a specific solar power reserve. MPPT is the underlying technique for the development of RPPT strategy as the main goal of MPPT is to extract maximum available solar power under changing environmental conditions like sunlight intensity and temperature. MPPT achieves maximum extraction by dynamically regulating the operating point of PV array to operate at their maximum power point. But grid stability is not ensured by the MPPT technique under varying solar power generation, leading to the development of the proposed DRPPT technique.

The proposed DRPPT technique dynamically controls the power output of PV plant that will be injected into the grid. The portion of output power that is not supplied to the grid is stored as a reserve power, which is used to support grid under grid disturbances. The grid voltage and frequency are deviated during grid disturbances, which are mainly stabilized by the proposed controller. In PV-grid power systems, the proposed model works in two stages: the boost DC-DC converter initially regulates the PV voltage to store reserve power levels and then the inverter converts the generated DC solar power into AC that will be injected into the grid. The stored power reserve is used to support grid adhering to recent standard grid codes such as IEEE 1547 and EN 50549, which enforce active power control, voltage regulation, and frequency support for grid-tied PV systems. Grid stability is ensured by the dynamic power curtailment and Fast Frequency Response (FFR) of the proposed model. The proposed controller is compatible with Supervisory Control and Data Acquisition (SCADA)-based control systems and smart inverters ensuring its integration into existing grid-compliant systems. The central SCADA system performs monitoring, data logging, and control, thereby improving the efficiency, reliability, and operational stability of the PV system. It mainly consists of data acquisition, network communication, data presentation, and control components. To ensure the bidirectional communication between the central SCADA system and the proposed controller, a Modbus TCP/IP communication protocol is used, which is widely adopted in grid-tied PV systems. In Modbus TCP/IP protocol, data is transmitted in a Modbus frame, composed of two data units: the Application Data Unit (ADU), which handles the communication layer, and the Protocol Data Unit (PDU), which contains the actual data that is to be transmitted. The DRPPT controller transmits real-time PV parameters including PV voltage, PV current, PV output power, reserve power level, and grid frequency to the SCADA system for monitoring, data logging, and supervisory analysis. The SCADA system transmits control commands to the proposed controller, such as reserve power setpoints and operating mode selection (MPPT/RPPT), ensuring effective supervisory control.

#### 3.1.1. Operational modes.

The three modes of RPPT algorithm are Constant power mode, Dynamic response mode and adjustable reserve targeting. In Constant power mode, RPPT algorithm develops constant output to the grid, saving a portion of PV capacity for sudden fluctuations in grid demand. In Dynamic reserve mode, if the grid frequency gets shifted from the nominal value, RPPT switches to this mode by releasing the reserve power to help bring back grid frequency back to normal level. In Adjustable reserve targeting, RPPT allows the operators to adjust reserve targets based on real time grid requirements or anticipated demand. RPPT algorithm gets voltage and power as inputs and sends reference voltage to voltage controller block to control the duty cycle of the DC – DC converter.

#### 3.1.2. Algorithm.

**Fig 2:**
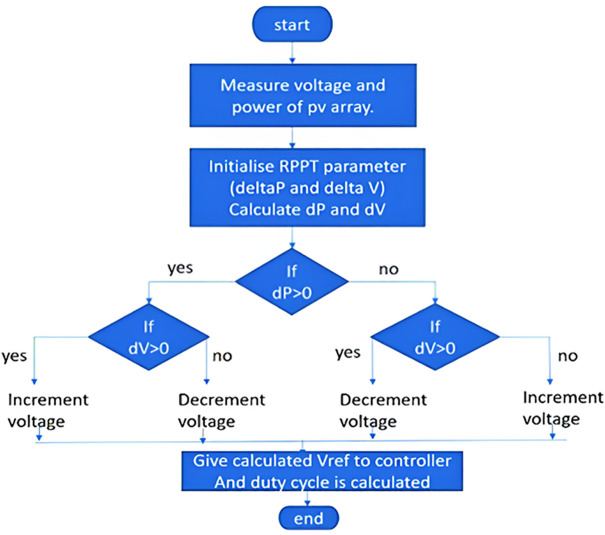
Proposed DRPPT algorithm.

The algorithm for DRPPT has been developed and its flow chart is given below in Fig 2. The algorithm is similar to the Perturb & Observe MPPT algorithm for optimizing the output power. It is started by calculating the PV array’s voltage and power then initializes parameters for power and voltage changes. The algorithm then calculates changes in power and voltage for the determination of the increment or decrement of the reference voltage. Finally, the corrected reference voltage is fed back to the controller, obtaining the duty cycle for maximum power extraction.

#### 3.1.3. Power reserve for frequency regulation.

Inertia response and power reserve are significant factors to minimize frequency deviations, thereby maintaining a tolerance of ±0.2 Hz from nominal frequency. This is necessary to avoid load shedding, generation loss, and system instability, as defined in the network.

In traditional synchronous machine-based power system, the inertia response is given by the kinetic energy of rotor to balance sudden load change. The power imbalance caused by the change in frequency is given by,


$$\begin{array}{c} \begin{array}{c} \Delta P=P_{m}-P_{e}=\text{2}Hf\left(\frac{df}{dt}\right) \end{array} \end{array}$$ (1)

Here, *H* is the inertia constant of the synchronous generator. However, in PV systems, mechanical inertia is absent as there is no rotor; therefore, the virtual inertia response is mathematically given as,


$$\begin{array}{c} \begin{array}{c} \Delta P_{vi}=\text{2}H_{vi}f\left(\frac{df}{dt}\right)=K_{i}\left(\frac{df}{dt}\right) \end{array} \end{array}$$ (2)

Here, $H_{vi}$ is the virtual inertia constant. Classical generators make use of governors for droop control mechanism, that is, regulation of power with respect to frequency changes. This droop control is also performed in solar power systems and the power change because of droop control is derived as,


$$\begin{array}{c} \begin{array}{c} \Delta P_{d}=-\left(\frac{\text{1}}{R}\right)\Delta f=K_{d}\Delta f \end{array} \end{array}$$ (3)

The droop coefficient $K_{d}$ is mathematically defined in Equation by assuming an initial pre-set power reserve $\Delta P_{\text{0}}$ and the total power reserve required for grid stability is given in Equation


$$\begin{array}{c} \begin{array}{c} K_{d}=\frac{\Delta P_{\text{0}}}{\Delta f}=\frac{\Delta P_{\text{0}}}{\left(\frac{\Delta f_{max}}{f_{\text{0}}}\right)} \end{array} \end{array}$$ (4)

$$\begin{array}{c} \begin{array}{c} \Delta P=\Delta P_{\text{0}}+\Delta P_{vi}+\Delta P_{d}=\Delta P_{\text{0}}+K_{i}\left(\frac{df}{dt}\right)+K_{d}\Delta f \end{array} \end{array}$$ (5)

**Fig 3:**
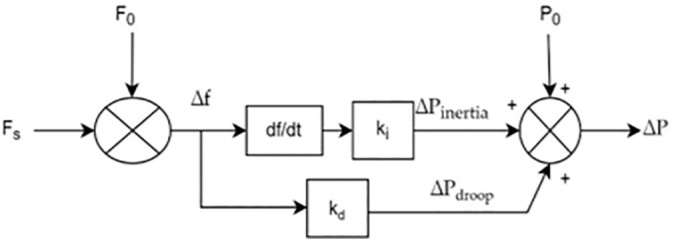
Block diagram of frequency regulation.

Based on this power reserve control, PV systems minimize their output power generation when grid frequency goes beyond the upper tolerance limit and increase their power generation when grid frequency falls behind the lower limit of tolerance. This dynamic power reserve control achieves stability of the grid, as portrayed in Fig 3.

#### 3.1.4. Point of operation of PV array.

Solar power systems use classical hill-climbing algorithmic techniques like Incremental Conductance (INC) and Perturb and Observation (P&O) methods to track MPP. Both methods operate on examining the relationship between solar panel’s power and voltage derivatives and have approximately equal tracking performance. However, INC technique is better than P&O as the computational complexity is reduced by the omission of second-order terms in the power equation. Moreover, P&O is less effective under quick change of irradiance levels. The INC model can be enhanced by incorporating a digital filter for fine-tuning the duty cycle of converter, thereby making the system to track MPP efficiently under changing conditions. Most standard grid laws mandate maximum power extraction, but it is not always suitable as injecting more power suddenly causes strain on the grid network, necessitating the requirement of a power reserve approach. This approach reduces the cost as it replaces traditional battery or supercapacitor-based energy storage systems.

The proposed DRPPT enhances the performance of the grid-connected PV models and can function both on DC–AC and DC–DC converters of the PV system. In the proposed mechanism, the controller reserves power by functioning on the DC-DC converter. Power can be reserved either by regulating voltage, power, or integrating methods like P&O and INC. In the proposed mechanism, the controller reserves power by controlling the power of the DC-DC converter.

**Fig 4:**
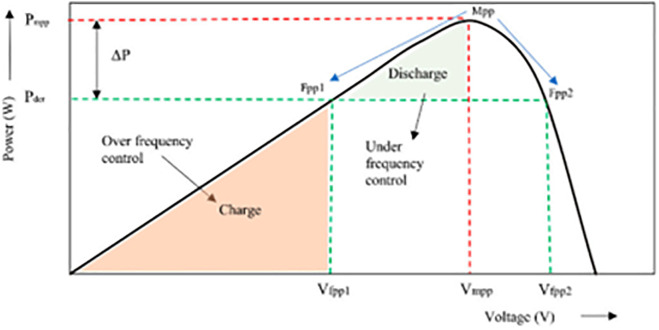
PV operating point characteristics for FPPT.

When PV arrays do not operate at the MPP, the operating region other than the MPP is known as FPPT mode. The PV arrays operating to the left of the MPP are known as Fpp1 and operating to the right of the MPP is known as Fpp2, as depicted in Fig 4. It is better for the system to work at Fpp1 region because the PV array operates at a slightly lower voltage than the Mpp, which enhances system stability. At Fpp1 region, voltage deviations can be detected earlier, more power reserve can be maintained, system response is achieved faster, conversion efficiency is improved, and overall grid stability is enhanced. Moreover, the lower operating voltage provides a larger stability margin and decreases the risk of voltage collapse during sudden irradiance changes. Even though the PV arrays operating in the Fpp2 region generates more power, stability is not guaranteed, as the system is more sensitive to disturbances. When the grid frequency increases beyond the nominal frequency, this over-frequency must be brought back to the nominal frequency to stabilize the grid. To achieve this, the FPPT control decreases the operating voltage of PV to reserve some amount of solar power. This stored reserve power will be discharged to grid when grid frequency decreases than the nominal frequency.

The voltage points set by the FPPT using P&O algorithm for power reserve control on both the right-hand and left-hand sides of MPP are given by Equations 6 and 7, respectively.


$$\begin{array}{c} \overset{+}{\underset{Xpp}{V}}=\left\{ \begin{array}{c} @lV_{mppt,}\;if\;P_{derated}\leq P_{MPP}\\ V_{PV}+V_{step},\;if\;P_{derated}>P_{MPP} \end{array}\right.\; \end{array}$$ (6)

$$\begin{array}{c} \overset{-}{\underset{Xpp}{V}}=\left\{ \begin{array}{c} @lV_{mppt,}\;if\;P_{derated}\leq P_{MPP}\\ V_{PV}-V_{step},\;if\;P_{derated}>P_{MPP} \end{array}\right.\; \end{array}$$ (7)

Here, $V_{step\;}$ denotes the perturbation step size utilized in the power reserve algorithm. *V*_*mppt*_ refers to the voltage at which maximum power is achieved, and *V*_*fpp*_ represents the regulated voltage derived from the derated PV system. The values of $V_{step\;}$, power reserve, and PV power output to the grid are predefined. To facilitate frequency regulation through this control strategy, the step size is adapted in response to frequency deviations.

## 4. Results

The proposed DRPPT including both MPPT and RPPT analysis is simulated in MATLAB and also validated in real-time hardware setup. For simulation, Soltech panel is utilized and the basic PV panel parameters are illustrated in Table 1. Both the hardware and simulation results are discussed in detail below.

**Table 1:** Basic PV panel parameters.

Parameters	Value
STC Power Rating $\left(P_{mp}\right)$	215W
PTC Power Rating ${(P}_{mp})$	189.4W
PTC/STC Power ratio	88.1%
Open circuit Voltage ${(V}_{oc})$	36.3V
Short Circuit current $\left(I_{sc}\right)$	7.84A
Current at Maximum Power ${(I}_{mp})$	7.35A
Voltage at Maximum Power $\left(V_{mp}\right)$	29 V
No of parallel cells $\left(N_{p}\right)$	10
No of series cell $\left(N_{s}\right)$	47
Voltage ripple (ΔV)	1%
Current ripple (ΔI)	5%
Switching frequency $\left(f_{s}\right)$	5KHz
Rated Power	100KW

The proposed DRPPT method changes the solar power reference $P_{pv}$-ref for regulating the power reserve, ΔP of the solar power plant. It can either take power reserve reference percentage $P_{ref}$% or $P_{pv}$-ref as inputs and the proposed controller will follow this reference and %P will vary in accordance with $P_{mpp}$. %P measures the ability of the proposed model to maintain the power reserve with respect to $P_{mpp}$, which is given mathematically as,


$$\begin{array}{c} \begin{array}{c} \%\Delta P=\frac{\Delta P}{Pmpp}\times \text{1}\text{0}\text{0} \end{array} \end{array}$$ (8)

**Fig 5:**
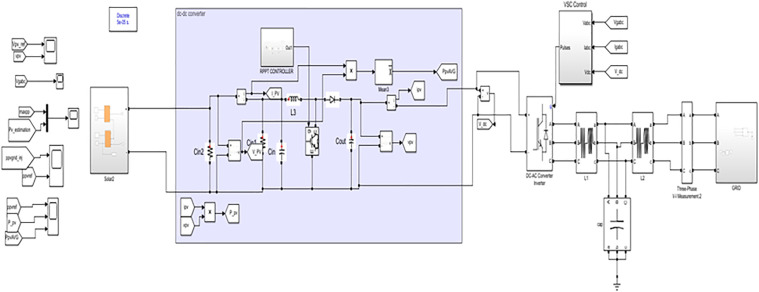
Simulation diagram.

**Fig 6:**
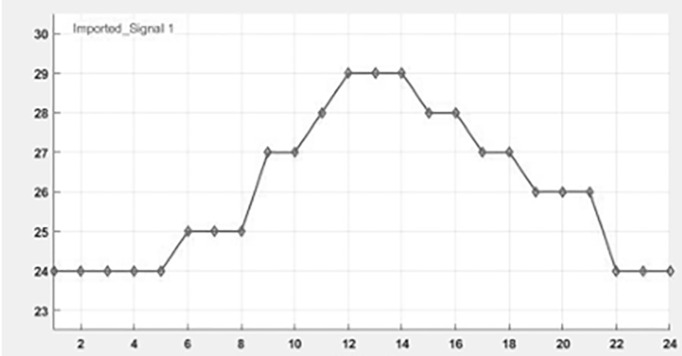
Temperature Vs time graph.

**Fig 7:**
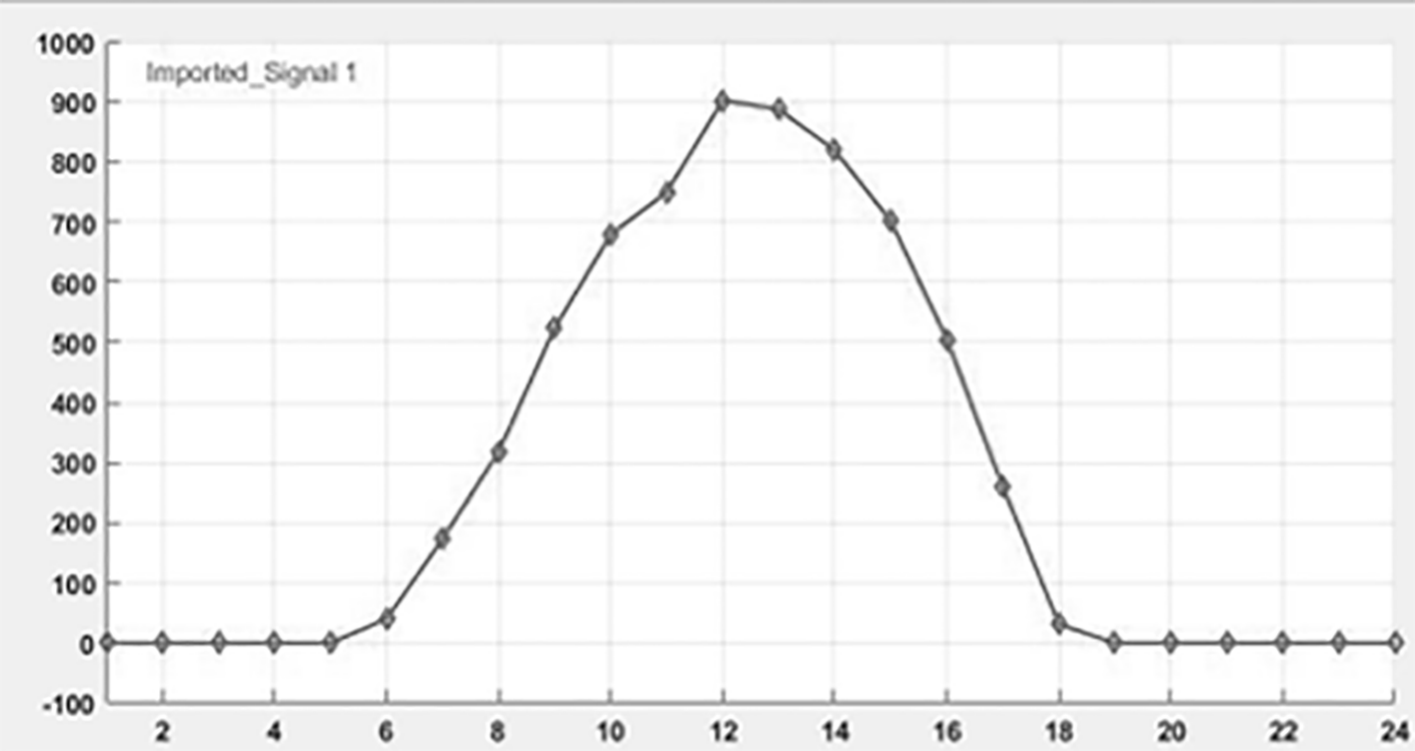
Irradiance Vs time graph.

The simulation model represents a PV power generation system using the proposed DRPPT algorithm for optimal conversion of energy. The PV array, being irradiance and temperature dependent, generates DC power, which further feeds the DC-DC boost converter. In this converter, the voltage is stepped up under the control of a reference voltage attained through the RPPT technique. The algorithm for RPPT technique calculates reference voltage on real-time inputs of PV voltage and power, in order to maximize the power output. This computed voltage is passed to the PV voltage controller, which regulates the converter’s duty cycle, D, for optimal performance. The boosted DC power is consequently converted into AC power, in a three-phase form, through an inverter for integration into the grid. This simulation evaluates the system’s efficiency and dynamic response under different insolation. The simulation connection of DRPPT implementation is shown in Fig 5. In real-world industrial PV plants, the MW-scale solar farms are composed of multiple PV strings connected to individual DC–DC converters and integrated through centralized inverters. Since the proposed DRPPT controller operates locally at the DC-DC converter level, it controls the PV voltage and reserve power level for each PV string independently. Therefore, the proposed DRPPT controller can be applied independently to each PV array without increasing computational complexity. This ensures scalability of the proposed DRPPT controller from kW-scale to MW-scale PV power plants. The temperature and irradiance input given for 24 hours is shown as a graph in Figs 6 and 7 respectively.

**Fig 8:**
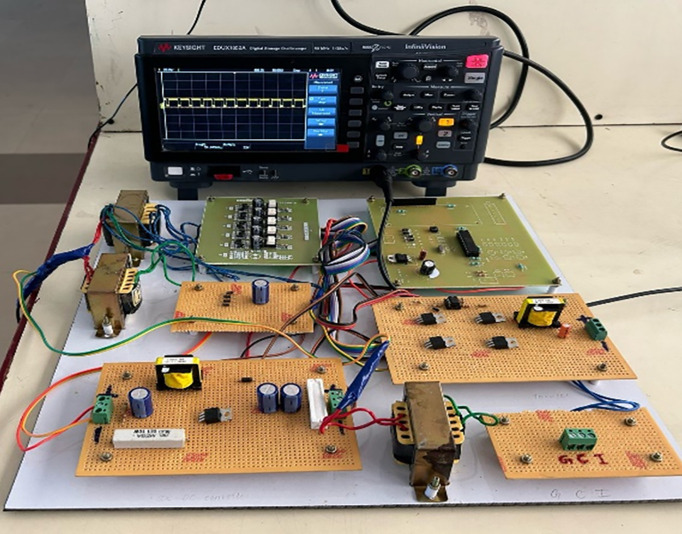
Hardware prototype.

Experimental verification of the proposed DRPPT algorithm is performed with a hardware prototype for a grid-connected PV system as shown in Fig 8. A dsPIC microcontroller is used as the main control unit to execute the RPPT algorithm, providing real-time power tracking and grid support capabilities. It handles real-time grid frequency changes and adapted the RPPT operation accordingly to meet grid stability standards. The power conversion stage included MOSFET switches in a DC-DC converter configuration for PV-side voltage regulation and a single-phase inverter for grid connection. In this present study, a controlled AC supply was utilized to emulate the PV output in place of real solar panel. The AC voltage is then converted into DC voltage by a bridge rectifier that corresponds to the PV power generation under varying conditions. This approach was adopted due to practical constraints associated with indoor laboratory testing, including the unavailability of controlled solar irradiance conditions and the need for repeatable and stable input characteristics. The rectified source allowed consistent evaluation of the proposed control strategy and ensured safe and reliable experimental operation.

**Fig 9:**
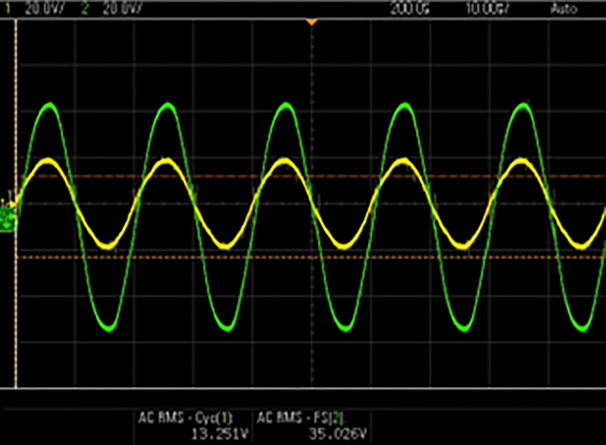
Grid synchronization.

Fig 9 depicts the synchronization process of grid happening between power grid and inverter output. To effectively synchronize the inverter output with the grid, a Zero-Crossing Detector (ZCD) is employed, which determines the accurate phase alignment between inverter output and grid voltage. ZCD then sends synchronization pulses to the inverter controller followed by the efficient power injection into the grid, ensuring stable and reliable grid operation without the occurrence of any disturbances.

**Fig 10:**
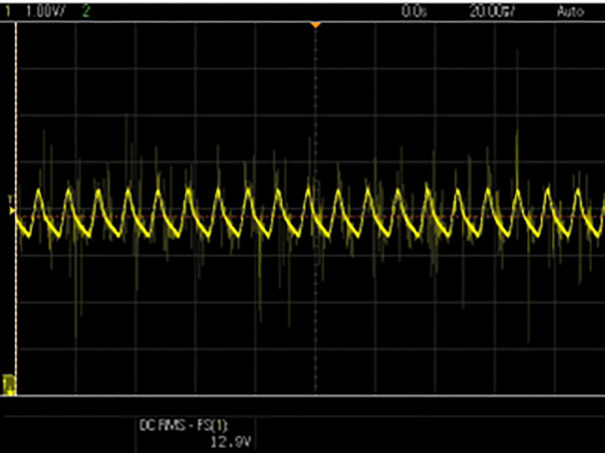
DC-DC converter input.

**Fig 11:**
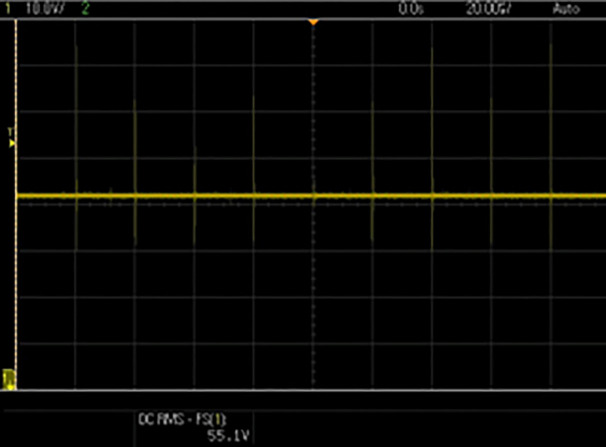
DC-DC converter output.

**Fig 12:**
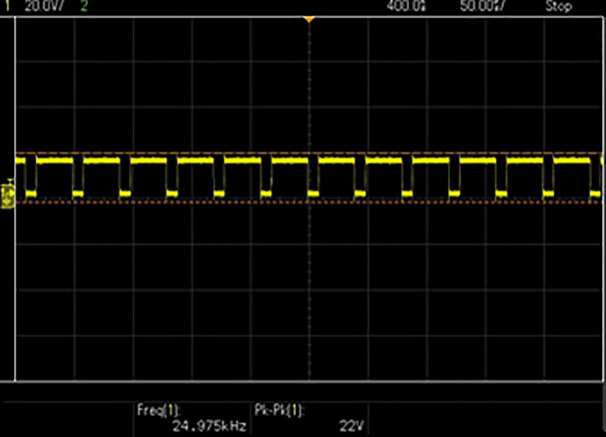
MOSFET gate pulse.

Fig 10 shows the stable and ripple-free DC input waveform of the DC–DC converter and Fig 11 displays the output voltage waveform of the DC–DC converter after being processed by the proposed controller. The converter regulates the output voltage according to $V_{pv-ref}$ generated by the proposed algorithm. The stable output waveform indicates the effectiveness of the duty-cycle control provided in Fig 12, ensuring the inverter gets the appropriate DC voltage for grid injection.

Fig 12 portrays the PWM gate signals produced by the dsPIC microcontroller for to be given to the MOSFET devices of the DC–DC converter. The figure displays changing duty cycle, which clearly shows the real-time adjustments made by the proposed controller to maintain the optimal current or voltage under varying irradiance and operating conditions. This ensures stable and efficient operation of the main utility grid.

**Fig 13:**
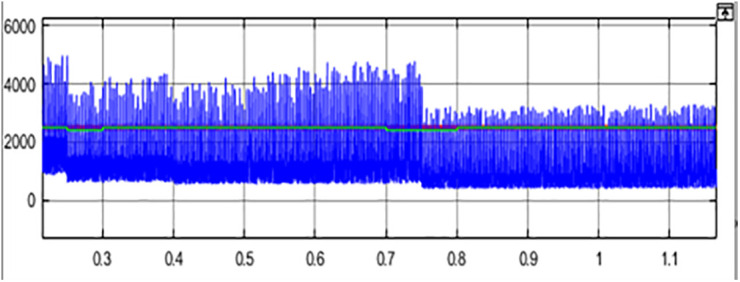
PV Power Vs PV Power Reference Vs Average PV Power.

**Fig 14:**
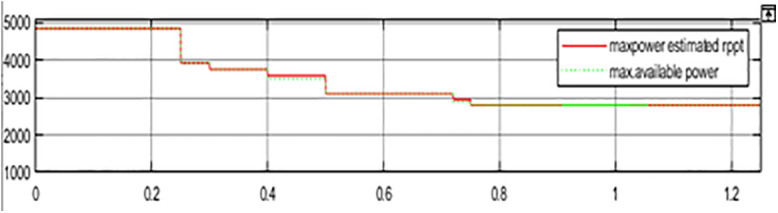
Maximum power obtained and available power.

**Fig 15:**
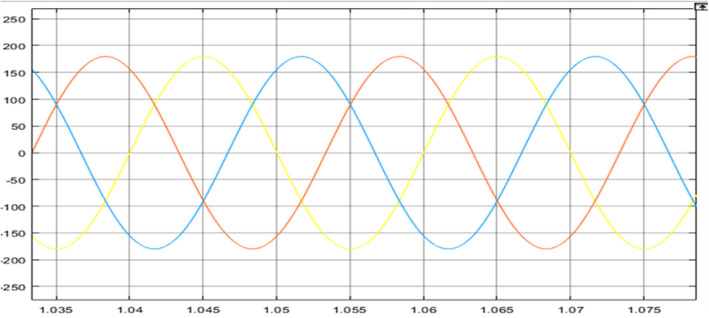
Output voltage waveform.

**Fig 16:**
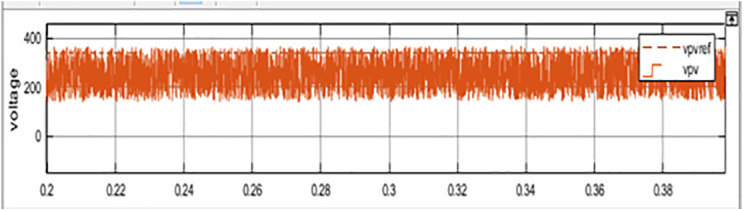
Voltage reference and PV power.

**Fig 17:**
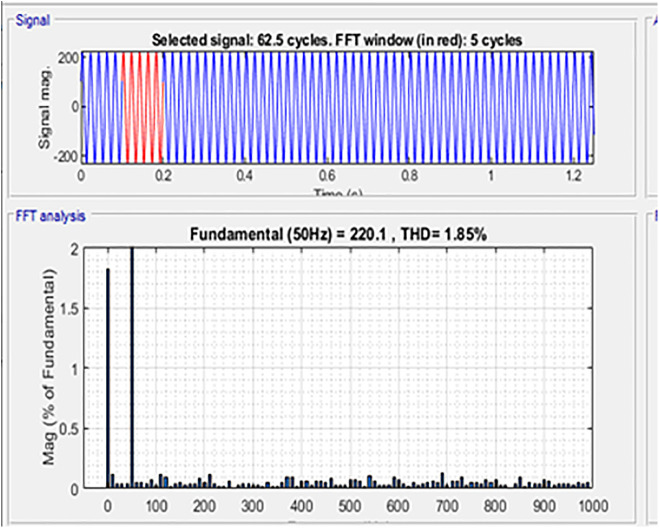
THD value.

The comparison between PV Power, PV Power Reference and Average PV Power is shown in Fig 13. The maximum power obtained, and the estimated PV power is shown in Fig 14. The generated power is integrated with the grid using an inverter. Fig 15 represents three phase voltage waveforms (output voltage waveform of the inverter) supplied to the grid. The reference voltage and PV voltage are shown in Fig 16. The THD of grid side is 1.85% and it is shown in Fig 17.

**Fig 18:**
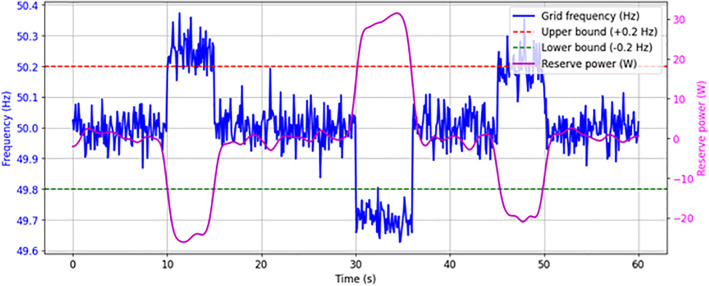
Grid frequency regulation.

Fig 18 depicts the attainment of grid stability through grid frequency regulation by the proposed DRPPT controller. When the grid frequency goes below the lower tolerance grid frequency limit, the proposed controller releases the reserve power to grid to restore its nominal frequency. On the other hand, when the grid frequency goes above the upper tolerance limit, the proposed controller reserves some amount of PV power to maintain grid frequency at nominal value.

Table 2 presents the techno-economic comparison between battery reserve systems and the no-battery power systems. Battery systems require high initial investment costs along with periodic maintenance and replacement due to battery degradation. However, no-battery power systems including the existing techniques such as RPPT [17], GFPPT [18] and GA-based FPPT [19], and the proposed DRPPT controller make use of the existing PV control to maintain reserve power through controlled PV curtailment, resulting in no additional storage infrastructure and reduced cost. In addition, no-battery systems show superior technical performance with high efficiency and reduced energy losses.

**Table 2:** Techno-economic comparison.

Parameters	Techniques				
	Battery reserve	RPPT [17]	GFPPT [18]	GA-based FPPT [19]	Proposed DRPPT
Investment cost (US$/kWh)	177	0	0	0	0
Maintenance cost (US$/kWh/year)	50	Negligible	Negligible	Negligible	Negligible
Replacement cost (US$/kWh)	150	Not required	Not required	Not required	Not required
Lifetime (years)	5-10	20-25	20-25	20-25	20-25
Efficiency (%)	87-92	≈100 (no storage conversion losses)	≈100 (no storage conversion losses)	≈100 (no storage conversion losses)	≈100 (no storage conversion losses)
Energy loss (%)	8-13 (storage loss)	0.73	0.61	0.47	0.32

**Fig 19:**
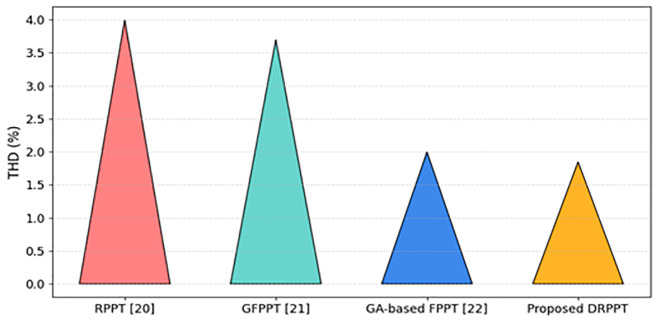
THD value comparison of different algorithms.

The comparative analysis of the THD values for different techniques is provided in Fig 19. The proposed DRPPT algorithm has THD of 1.85% showing improved ancillary THD suppression performance. In contrast, the conventional RPPT [17], GFPPT [18], and GA-based FPPT [19] techniques have higher THD values of 4%, 3.7%, and 2%, respectively, indicating poor grid quality power generation. The results clearly tell that the proposed DRPPT controller reduces THD by 53.75%, 50%, and 7.5% compared to RPPT [17], GFPPT [18], and GA-based FPPT [19] techniques. This indicates that the proposed DRPPT controller achieves superior harmonic mitigation, thereby enhancing grid injected power quality and adhering to IEEE harmonic grid standards.

**Fig 20:**
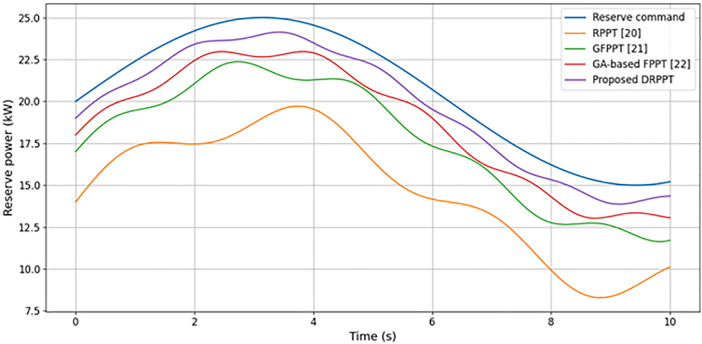
Comparative analysis of reserve power tracking performance.

Fig 20 presents the comparative analysis of reserve power tracking performance of different techniques. Conventional control algorithms such as RPPT [17], GFPPT [18], and GA-based FPPT [19] exhibit high-frequency oscillations and large deviations, representing limited reserve power following ability. The RPPT [17], GFPPT [18], and GA-based FPPT [19] techniques show peak-to-peak oscillations of 2 kW, 1 kW, and 0.7 kW, respectively, indicating significant fluctuations in reserve power tracking. However, the proposed DRPPT model tracks the reserve power command in close approximation, achieving peak-to-peak oscillation of 0.4 kW, thereby providing a smoother response with minimal error. This exhibits the improved and stable adaptive capability of the proposed model with high tracking accuracy owing to its adaptive decision rules. By closely following the reserve command and maintaining a flexible reserve, the proposed DRPPT algorithm is able to quickly absorb or inject absorb power, thereby stabilizing the grid.

**Fig 21:**
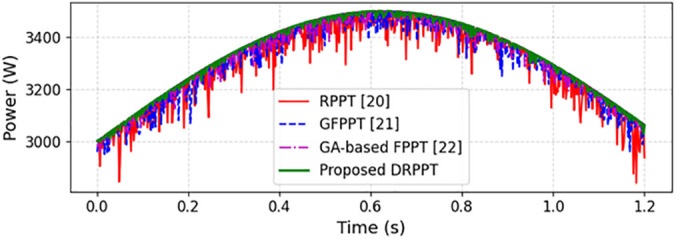
Comparative analysis of power output for various models.

Fig 21 depicts the comparative assessment of power output profiles of existing and proposed models. The proposed DRPPT algorithm adheres to grid requirements and has the ability to sustain stable output power with minimal fluctuations. However, the existing techniques have high ripples associated with them illustrating that the proposed model balances reliability, stability, and efficiency for grid-connected PV applications. Even under high penetration solar power, the proposed model works well by generating output power with minimal oscillations, thereby enhancing grid stability.

**Fig 22:**
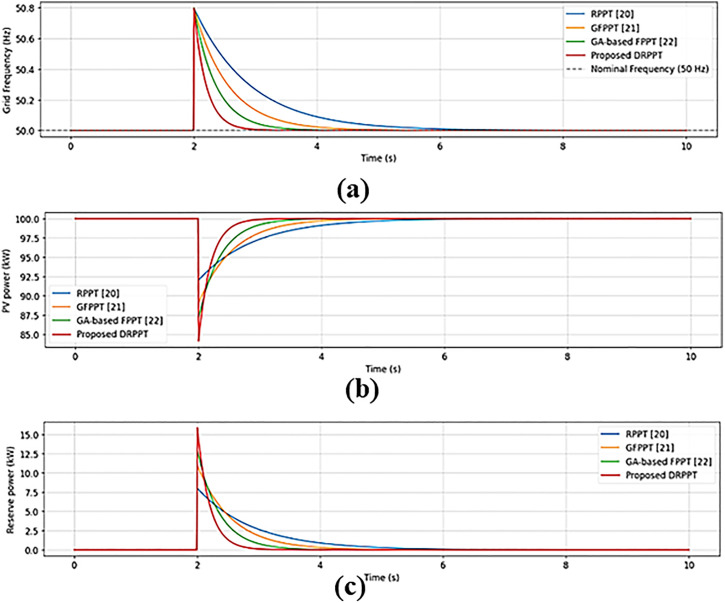
Comparative analysis of different techniques in frequency stabilization: (a) Frequency response; (b) PV reference power; and (c) Reserve power.

Fig 22 shows the comparison of different techniques in achieving grid frequency stabilization through reserve power determination and PV power estimation. Fig 22a shows the frequency response, Fig 22b shows the PV power, and Fig 22c shows the reserve power profile. At 2 seconds, the grid frequency is deviated from the nominal frequency due to power demand and power production mismatch. To stabilize the grid frequency, RPPT [17], GFPPT [18] GA-based FPPT [19], and the proposed DRPPT controllers decrease the PV operating voltage from the maximum operating power 100 kW and the curtailed power is stored as a reserve. This reserve power will be used when grid frequency becomes lower than the nominal frequency. Of all the techniques, the proposed controller stabilizes the grid faster because of its effective reserve power management, thereby resulting in improved frequency stabilization performance.

## 5. Conclusion

In this research, a novel DRPPT algorithm is proposed to inject flexible power into the grid while maintaining stability in the grid. Through the proposed DRPPT algorithm, the power output of the solar panel system is dynamically controlled to store desired solar power reserve level. The proposed model is simulated in MATLAB, and the simulation results exhibit its capability to respond effectively to changing irradiance levels and temperature. Moreover, the results demonstrated the effective voltage and frequency regulation of the proposed controller. The grid stability is enhanced without the employment of energy storage devices, resulting in cost-effective systems. The proposed model achieved less THD value as mandated by the grid standards, which is nearly 53.75%, 50%, and 7.5% less compared to conventional RPPT, GFPPT, and GA-based FPPT techniques, respectively. It is a promising solution to modern grid-connected solar systems with their dynamic power reserve capability. The proposed algorithm is also implemented on a small prototype hardware, and its effectiveness has been validated. Overall, the proposed DRPPT controller provides a reliable and adaptive solution for maintaining grid stability, improving power quality, and ensuring rapid response to dynamic grid conditions, making it highly suitable for modern grid-integrated solar energy systems. In the future, the same can be validated on large scale real PV systems under diverse operating conditions, including partial shading and stochastic irradiance variations to enhance the adaptability of the grid-tied solar systems.

## References

[1] Motahhir S, El Hammoumi A, El Ghzizal A. The most used MPPT algorithms: review and the suitable low-cost embedded board for each algorithm. J Clean Prod. 2020;246:118983. doi: 10.1016/j.jclepro.2019.118983

[2] Sadick A. Maximum power point tracking simulation for photovoltaic systems using perturb and observe algorithm. Solar Radiation-Enabling Technologies, Recent Innovations, and Advancements for Energy Transition. IntechOpen; 2023.

[3] Manoharan P, Subramaniam U, Babu TS, Padmanaban S, Holm-Nielsen JB, Mitolo M, et al. Improved perturb and observation maximum power point tracking technique for solar photovoltaic power generation systems. IEEE Syst J. 2021;15(2):3024–35. doi: 10.1109/jsyst.2020.3003255

[4] Abo-Sennah MA, El-Dabah MA, Mansour AE-B. Maximum power point tracking techniques for photovoltaic systems: a comparative study. Int J Elect Comput Eng. 2021;11(1):57. doi: 10.11591/ijece.v11i1.pp57-73

[5] Haghighat M, Niroomand M, Dehghani Tafti H, D. Townsend C, Fernando T. A Review of state-of-the-art flexible power point tracking algorithms in photovoltaic systems for grid support: classification and application. J Modern Power Syst Clean Energy. 2024;12(1):1–21. doi: 10.35833/mpce.2022.000845

[6] Reddy MKK, Sarkar V. The quantum‐mode regulated power point tracking in a photovoltaic array for application under the quantised converter duty ratio. IET Renew Power Gen. 2021;15(8):1748–64. doi: 10.1049/rpg2.12143

[7] Javed S, Ishaque K. A comprehensive analyses with new findings of different PSO variants for MPPT problem under partial shading. Ain Shams Eng J. 2022;13(5):101680. doi: 10.1016/j.asej.2021.101680

[8] Ambe H, Feudjio C, Raoul Fotso Mbobda C, Alombah NH. A new framework for improving MPPT algorithms through search space reduction. Result Eng. 2024;22.

[9] Narang A, Farivar GG, Dehghani Tafti H, Ceballos S, Pou J, Townsend CD, et al. Dynamic Flexible Power Point Tracking in Photovoltaic Power Plants. In: IECON 2021 – 47th Annual Conference of the IEEE Industrial Electronics Society. Toronto, ON, Canada; 2021. pp. 1–6. doi: 10.1109/iecon48115.2021.9589818

[10] Narang G, Farivar HD, Tafti J, Pou J. Power reserve control methods for grid-connected photovoltaic power plants: A review. In: 2022 IEEE 7th Southern Power Electronics Conference (SPEC). Nadi, Fiji; 2022.

[11] Saxena V, Kumar N, Singh B, Panigrahi BK. A rapid circle centre-line concept-based MPPT algorithm for solar photovoltaic energy conversion systems. IEEE Trans Circuits Syst I. 2021;68(2):940–9. doi: 10.1109/tcsi.2020.3038114

[12] Maaz N, Mekhilef S, Mubin M, Ahmed S, Seyedmahmoudian M, Stojcevski A, et al. Advancements in flexible power point tracking and power control strategies for photovoltaic power plants: A comprehensive review. Energy Rep. 2024;12:237–50.

[13] Sharma S, Jately V, Kuchhal P, Kala P, Azzopardi B. A comprehensive review of flexible power-point-tracking algorithms for grid-connected photovoltaic systems. Energies. 2023;16(15):5679. doi: 10.3390/en16155679

[14] Liu Y-H, Chen G-J, Liu C-L, Su C-Y. Comprehensive review on fast maximum power point tracking algorithms for solar power generation systems. Ain Shams Eng J. 2024;15(12):103093. doi: 10.1016/j.asej.2024.103093

[15] Mahato TR, Choudhury B, Nayak SR. Biswal R, Dash R. Sensorless FPPT Tracking Mechanism based on Forecasting Data. 2022 1st IEEE International Conference on Industrial Electronics: Developments & Applications (ICIDeA). Bhubaneswar, India: 2022.

[16] Saxena V, Kumar N, Singh B, Panigrahi BK. A rapid circle centre-line concept-based MPPT algorithm for solar photovoltaic energy conversion systems. IEEE Trans Circuits Syst I. 2021;68(2):940–9. doi: 10.1109/tcsi.2020.3038114

[17] Narang A, Farivar GG, Tafti HD, Ceballos S, Beniwal N, Pou J, et al. Dynamic reserve power point tracking in grid-connected photovoltaic power plants. IEEE Trans Power Electron. 2023;38(5):5939–51. doi: 10.1109/tpel.2023.3240186

[18] Tafti HD, Wang Q, Townsend CD, Pou J, Konstantinou G. Global flexible power point tracking in photovoltaic systems under partial shading conditions. IEEE Trans Power Electron. 2022;37(9):11332–41. doi: 10.1109/tpel.2022.3167657

[19] Ouatman H, Boutammachte N-E. A genetic algorithm approach for flexible power point tracking in partial shading conditions. Result Eng. 2024;24.

[20] Zhong C, Zhou Y, Yan G. Power reserve control with real-time iterative estimation for PV system participation in frequency regulation. Int J Electric Power Energy Syst. 2021;124:106367. doi: 10.1016/j.ijepes.2020.106367

[21] Gomez-Merchan R, Vazquez S, Alcaide AM, Tafti HD, Leon JI, Pou J, et al. Binary search based flexible power point tracking algorithm for photovoltaic systems. IEEE Trans Ind Electron. 2021;68(7):5909–20. doi: 10.1109/tie.2020.2998743

[22] Pawar B, Batzelis E, Chakrabarti S, Pal B. Grid-forming control for solar PV systems with power reserves. IEEE Trans Sustain Energy. 2021;12(4):1947–59. doi: 10.1109/tste.2021.3074066

[23] Kumaresan A, Tafti HD, Kandasamy NK, Farivar GG, Pou J, Subbaiyan T. Flexible power point tracking for solar photovoltaic systems using secant method. IEEE Trans Power Electron. 2021;36(8):9419–29. doi: 10.1109/tpel.2021.3049275

[24] Chen S, Zhu L, Conejo AJ, Wei Z. Unit commitment of integrated electric-natural gas-hydrogen systems including FCR services. IEEE Trans Power Syst. 2026;:1–12. doi: 10.1109/tpwrs.2026.3653968

[25] Feng J, Ren Z, Li C, Li W. A benders-combined safe reinforcement learning framework for risk-averse dispatch considering frequency security constraints. IEEE Trans Circuits Syst II. 2025;72(8):1063–7. doi: 10.1109/tcsii.2025.3584894

[26] Fei Z, Zou Y, Hua W, Banda OV, Guerrero JM, Li Z. Weather routing-based multi-energy ship microgrid operation under diverse uncertainties: a risk-averse stochastic approach. IEEE Trans Smart Grid. 2025;16(6):4648–59. doi: 10.1109/tsg.2025.3604590

